# Systemic Mastocytosis: A Rare Cause of Diarrhea

**DOI:** 10.7759/cureus.9112

**Published:** 2020-07-10

**Authors:** Leonard Hamera, Andrew M Santos, Sean-Patrick A Prince, Sreekanth Chandrupatla, Jeffrey Jordan

**Affiliations:** 1 Internal Medicine, Citrus Memorial Hospital, Inverness, USA; 2 Internal Medicine, HCA-USF Consortium, Citrus Memorial Hospital, Inverness, USA; 3 Gastroenterology, Citrus Memorial Hospital, Inverness, USA

**Keywords:** systemic mastocytosis, diarrhea, nausea and vomiting, fecal incontinence

## Abstract

Mastocytosis is a spectrum of neoplastic, clonal cell disorders that are characterized by mast cell hyperplasia and accumulation. Disease and clinical presentation can vary depending on the extent of spread, ranging from skin-limited cutaneous mastocytosis to systemic mastocytosis that can mimic other disease processes. Symptoms may include pruritus, flushing, hypotension, headaches, abdominal pain, nausea, vomiting, and diarrhea. Although gastrointestinal (GI) symptoms are present in a majority of patients with systemic disease, the actual percentage of gut mast cell infiltration remains unknown. Here we describe a case of diarrhea secondary to GI involvement of systemic mastocytosis. A 55-year-old woman with a known history of systemic mastocytosis and medical noncompliance complained of persistent chronic diarrhea for one year. She was evaluated for other causes of diarrhea but all additional testing was unrevealing. She ultimately underwent upper endoscopy and colonoscopy in which biopsy and histologic analysis confirmed the presence of mastocyte infiltration. She was restarted on her medical therapy and her symptoms resolved. In conclusion, systemic mastocytosis is an uncommon cause of chronic diarrhea. However, in select patients, it is important to obtain a thorough medical history and exclude other potential causes.

## Introduction

Mastocytosis refers to a group of disorders characterized by excessive mast cell accumulation in one or multiple tissues [1]. It can be simplified into two groups: cutaneous mastocytosis (CM) and systemic mastocytosis (SM). The World Health Organization (WHO) outlined criteria to appropriately diagnose and classify mastocytosis. CM is now divided into maculopapular CM/urticaria pigmentosa, diffuse cutaneous mastocytosis, and localized mastocytoma of skin. While SM can be classified into indolent SM, aggressive SM, smoldering SM, SM with an associated hematologic neoplasm or mast cell leukemia [2].

The exact incidence is unknown. Mastocytosis has been shown to affect males and females equally, however, there is a slight male predominance in childhood and a slight female predominance in adulthood [3]. Additionally, adults are more often found to have systemic forms of the disease with cutaneous forms accounting for less than 5% of adult cases [4].

The pathogenesis of all forms of mastocytosis results from the combination of chronic and episodic mast cell-mediator release with excessive mast cell accumulation in one or more tissues. Mast cells contain a variety of vasoactive mediators that generate an inflammatory response. Contributing mediators include histamine, heparin, leukotriene, prostaglandin, platelet activating factors, protease, and cytokines such as tumor necrosis factor (TNF). The signs and symptoms associated with sudden and extensive mass cell mediator release are similar to those associated with allergic and/or anaphylactic reactions. Although the molecular abnormalities are not completely understood, mutations in KIT activity and nonactivating mutations of KIT have been implicated in the pathogenesis of both CM and SM [1]. The stem-cell factor also called KIT (CD117) ligand is a growth factor that is essential for normal development and expansion of mast cells from hematopoietic progenitor. Mast cells express receptors for the receptor tyrosine kinase KIT on their surface. Although many of the molecular defects associated with mastocytosis involve gain-of-function mutations in KIT, there is no consistent correlation between the type of mutation and either phenotype or prognosis [5]. More than 95% of adults with SM have an exon 17 KIT mutation, most commonly D816V [6]. The precise mechanism by which KIT activating mutation enhances signal is not fully characterized, but the effects lead to independent stem cell factor activation. Clonal expansion and apoptotic defects of the KIT mutated mast cells are thought to provide the basis for the pathological accumulation of mast cells in tissue [7]. There are many observed mutations but the most common mutation detected in human mast cell disease occurs in the codon 816 and consists of a substitution of valine for aspartate [6]. Additionally, rare familial cases of mastocytosis have been reported, with recognized KIT mutations demonstrated in some [8].

Systemic mastocytosis involves extracutaneous site(s) with commonly reported sites including the bone marrow and gastrointestinal (GI) tract [9]. Mastocytosis will present with symptoms of mediator release, with GI dysfunction being observed in both CM and SM [10]. These GI symptoms including abdominal pain, diarrhea, nausea, vomiting, peptic ulcer disease, and GI bleed can be precipitated by the same triggers that cause systemic symptoms. Abdominal pain and diarrhea are reported in up to 80% of patients with SM, with duodenal ulceration and severe duodenitis in 30%-50% of untreated cases [11-12]. Diarrhea can result from increased motility induced by PGD2 secretion. Histamine and other mediators released from local and distant mast cells increase gastric acid secretion. Serum histamine concentration may also be elevated, particularly in the setting of ulcer disease, suggesting that circulating histamine contributes to the basal acid hypersecretion [10, 12]. Malabsorption and subsequent weight loss can result from extensive mucosal and submucosal infiltrations with mast cells.

## Case presentation

A 55-year-old Caucasian female was seen and evaluated by outpatient gastroenterology for persistent chronic diarrhea. Diarrhea was described as four to six liquid brown bowel movements a day for the past year. Occasionally some solid foods pass whole in her stools. The episodes woke her from sleep and she had three episodes of fecal incontinence during this time. She denied triggers for events. She endorsed associated intermittent diffuse cramping abdominal pain with sporadic episodes of nausea and vomiting. Additionally, the patient admitted to fatigue, loss of appetite, weight loss, and itching. She denied fevers or chills, diaphoresis, dysphagia, jaundice, excessive belching or flatulence, hematemesis, hematochezia, or melena. Her last colonoscopy and endoscopy were both taken 10 years ago. Past medical history included hepatitis C treated successfully with sofosbuvir-velpatasvir, liver cirrhosis, esophageal varices, Barrett esophagus, systemic mastocytosis, pancytopenia, medical therapy noncompliance, and alcoholism. She had no previous surgeries. Family history was pertinent for colon cancer diagnosed in advanced age in her mother. The patient was a former smoker and denied any history of recreational or intravenous drug use. She was employed locally and had no history of travel outside of the area. Physical exam revealed an enlarged liver 3 cm below the costal margin. Pertinent negative findings included no evidence of jaundice, rashes, excoriations, scleral icterus, oral-pharyngeal abnormalities, lymphadenopathy, abdominal tenderness, swelling, or additional organomegaly.

**Figure 1:**
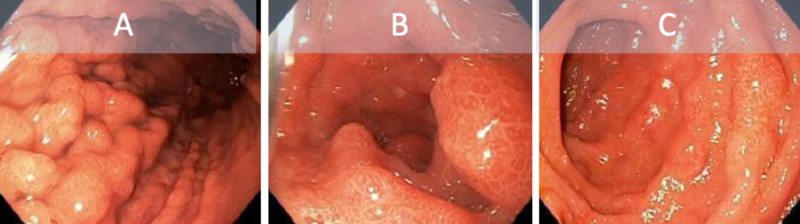
Endoscopy showing diffuse inflammatory and nodular changes to the gastric antrum (A) and duodenum (B and C).

**Figure 2:**
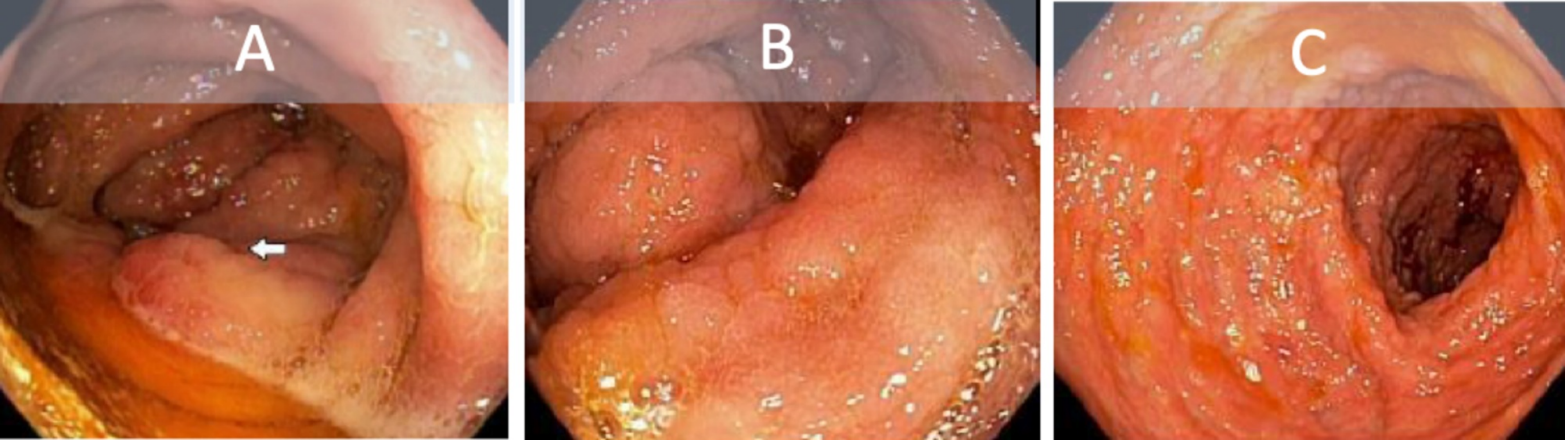
Colonoscopy showing diffuse inflammatory and nodular changes to the ileocecal valve (A), ascending colon (B), and transverse colon (C).

A review of her medications was unremarkable for laxatives, however, the patient stated she was not taking her midostaurin as prescribed. A complete blood count was notable for chronic pancytopenia and a complete metabolic panel showed hyperbilirubinemia and elevated liver enzyme function. Stool culture, Clostridium difficile screening, stool ova and parasite, fecal elastase, alpha fetal protein additionally were all unremarkable. She was subsequently taken for endoscopy and colonoscopy. Diffuse mucosal thickening and nodular changes were seen on endoscopy and colonoscopy (Figures 1-2). Histology was remarkable for fibrosis and chronic inflammatory changes of the stomach antrum and body, small bowel, right colonic, left colonic, and rectal mucosa, involved by mastocytes and consistent with systemic mastocytosis. Neoplastic markers BCL2, CDX2, pancytokeratin, CD10, CD20, CD5, CyclinD-1, and CD23 were negative. Findings were discussed with the patient at length and she ultimately decided to continue care and adhere to medical therapy. Her diarrhea resolved without further incidence to date.

## Discussion

Gastrointestinal manifestations of SM such as nausea, vomiting, and diarrhea are not uncommon. Chronic diarrhea specifically requires an in-depth investigation and determining mastocytosis as the cause of such symptoms may prove challenging. Exclusion of parasitic or bacterial organisms is a necessary step if indicated. Endoscopy and colonoscopy should be obtained at the digression of the treating provider to rule out alternative or additional pathology. Commonly, endoscopic features in SM patients are peptic esophagitis, peptic ulcers, thickened gastric or duodenal folds, nodular mucosa, ulcerative lesions, and dilated bowel loops [13-14]. In our patient, diffuse thickening and nodular changes were seen grossly and mastocytosis was present on mucosal biopsy.

**Figure 3:**
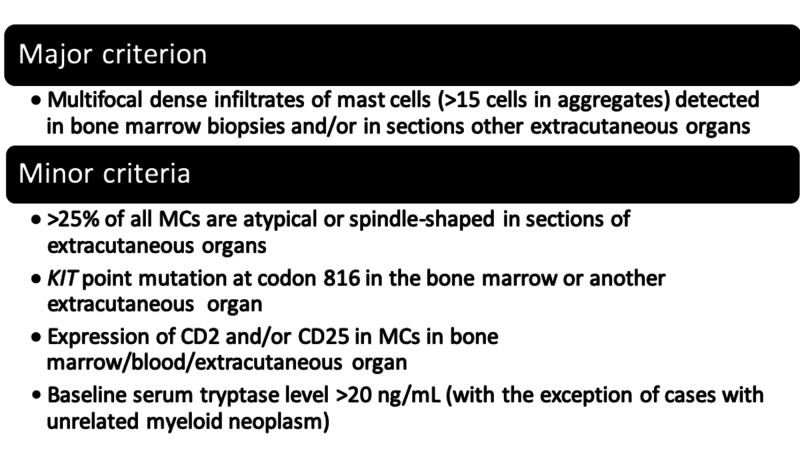
World Health Organization Diagnostic Criteria for SM. SM, systemic mastocytosis

Currently there is no consensus about GI biopsy criteria, but involvement should be highly suspected in patients with an established diagnosis of mastocytosis. Based on the WHO criteria, diagnosis of SM can be made when the major criterion and one minor or at least three minor criteria are present (Figure 3) [1]. These criteria are useful for supporting the diagnosis of SM for a patient with GI complaints.

Due to the rarity of SM, there is no standardized treatment regime. Patients often use a single agent or combination of medications for treatment. Literature on current treatment commonly describes midostaurin, cladribine, and allogenic hematopoietic cell transplant (HCT) for SM and its variants, with the latter as a potential curative therapy [15-17]. Midostaurin is a KIT inhibitor that has shown promise in patients with advanced SM and mast cell leukemia variations. Gotlib and colleagues reported a clinically meaningful response in most of their group with an added overall median survival benefit [15]. Although nausea, vomiting, and diarrhea are known side effects, patients in this study reported an overall quality of life improvement. Other pharmacotherapies, such as cladribine, work to reduce the mast cell burden. Cladribine is a synthetic purine analog capable of inducing apoptosis in mast cells [16]. In a study by Barete et. al., the overall response rate to treatment was around 50%, with indolent SM showing a greater response than more advanced forms of SM. Of note, there was a high degree of relapse in this patient population. HCT may be curative in a select population, however, patients must meet inclusion criteria, and in general should be relatively healthy [17]. It should be noted that there are currently no randomized controlled trials for these options and thus further research is needed to evaluate their efficacy. Given the side effect profiles potential conflicts arise when exploring how best to treat GI symptoms of SM. Inadequate treatment response may be mistaken for side effect and vice versa. As our patient was noncompliant with her therapy, the benefit of restating midostaurin outweighed these risks. Furthermore, there are no validated treatments for GI symptoms of SM. One case report cited patient improvement with a combination of H1 and H2 blocking agents [18]. Additionally, management should include hydration with enteral, or if needed IV fluids and electrolyte monitoring. Given an unpredictable relapse rate, patients should be educated on the importance of medication compliance, as noncompliance may complicate diagnosis and treatment.

Prognosis for SM is, in general, influenced by the disease state. In a review by the European Competence Network on mastocytosis, factors that are suggestive of a poorer prognosis include advanced disease states including associated malignancy and certain generic variants [19]. Prognostic models such as the Mayo Clinic, mutation-adjusted risk score (MARS), and Mayo-Hybrid models include additional factors such as age, elevated alkaline phosphatase level, cytopenia, and certain mutations. To our knowledge there is no literature connecting GI symptomatology such as nausea, vomiting, and diarrhea to a poorer prognosis. Our patient, however, has added comorbidities that may influence her overall prognosis. It is reasonable to consider that SM combined with liver cirrhosis, Barrett esophagus, and continued alcohol use places her in a poorer prognostic category, particularly given Barrett’s known risk of conversion to malignancy itself.

## Conclusions

Systemic mastocytosis is an uncommon cause of GI disease and patients can go undiagnosed for months to years. A high index of suspicion should be maintained for this subset of patients not meeting criteria for other disorders. Early invasive testing should be pursued as histologic analysis can lead to diagnosis. Once diagnosed, treatment mainly focuses on the disease subtype. At this time further investigation is needed to standardize a specific treatment for GI symptoms. 
